# Radiological Prediction of Isocitrate Dehydrogenase (IDH) Mutational Status and Pathological Verification for Lower-Grade Astrocytomas

**DOI:** 10.7759/cureus.27157

**Published:** 2022-07-22

**Authors:** Hirohito Yano, Yuka Ikegame, Kazuhiro Miwa, Noriyuki Nakayama, Takashi Maruyama, Soko Ikuta, Kazutoshi Yokoyama, Yoshihiro Muragaki, Toru Iwama, Jun Shinoda

**Affiliations:** 1 Department of Neurosurgery, Chubu Medical Center for Prolonged Traumatic Brain Dysfunction, Chubu Neurorehabilitation Hospital, Minokamo, JPN; 2 Department of Clinical Brain Sciences, Gifu University Graduate School of Medicine, Gifu, JPN; 3 Department of Neurosurgery, Central Japan International Medical Center, Minokamo, JPN; 4 Department of Neurosurgery, Gifu University Graduate School of Medicine, Gifu, JPN; 5 Department of Neurosurgery, Tokyo Women’s Medical University, Tokyo, JPN

**Keywords:** isocitrate dehydrogenase, pet, methionine, mrs, mri, astrocytoma

## Abstract

Background and objective

The isocitrate dehydrogenase (IDH) status of patients with World Health Organization (WHO) grade II or III astrocytoma is essential for understanding its biological features and determining therapeutic strategies. This study aimed to use radiological analysis to predict the IDH status of patients with lower-grade astrocytomas and to verify the pathological implications.

Methods

In this study, 47 patients with grade II (17 cases) or III astrocytomas (30 cases), based on 2016 WHO Classification, underwent methionine (MET) positron emission tomography (PET) and magnetic resonance spectroscopy (MRS) on the same day between January 2013 and June 2020. The patients were retrospectively assessed. Immunohistochemistry showed 23 cases of IDH-mutant and 24 of IDH-wildtype. Based on fluid-attenuated recovery inversion (FLAIR)/T2 imaging, three doctors blinded to clinical data independently allocated 18 patients to the clear boundary group between the tumor and the normal brain and 29 to the unclear boundary group. The peak ratios of N-acetylaspartate (NAA)/creatine (Cr), choline (Cho)/Cr, and Cho/NAA and the tumor-to-normal region (T/N) ratio for maximum accumulation in MET-PET were calculated. For statistical analysis, Fisher’s exact test was used to assess associations between two variables, and the Mann-Whitney U test to compare the values between the IDH-wildtype and IDH-mutant groups. The optimal cut-off values of MET T/N ratio and MRS parameters for discriminating IDH-wildtype from IDH-mutant were obtained using receiver operating characteristics curves.

Results

The unclear boundary group had significantly more IDH-wildtype cases than the clear boundary group (P<0.001). The IDH-wildtype group had significantly lower Cho/Cr (<1.84) and Cho/NAA (<1.62) ratios (P=0.02 and P=0.047, respectively) and a higher MET T/N ratio (>1.44, P=0.02) than the IDH-mutant group. The odds for the IDH-wildtype were 0.22 for patients who fulfilled none of the four criteria, including boundary status and three ratios, and 0.9 for all four criteria.

Conclusions

These results suggest that the combination of MRI, MRS, and MET-PET examination could be helpful for the prediction of IDH status in WHO grade II/III gliomas.

## Introduction

Since the 2016 World Health Organization (WHO; Geneva, Switzerland) Classification of tumors incorporated molecular diagnosis, the patients' isocitrate dehydrogenase (IDH) status has been clinically meaningful. For astrocytoma, the median survival of IDH-mutant (mut) and IDH-wildtype (wt) is 9.3 years and 1.9 years, respectively [1]. It was reported that IDH-mut WHO grade II astrocytomas showed sharp borders on T2-weighted imaging (T2WI), and they are predominantly located in a single lobe with less contrast enhancement [2,3]. In contrast, IDH-wt WHO grade II gliomas have a greater tumor volume and are located in combined lobes with an infiltrative pattern on magnetic resonance image (MRI) [3,4]. On the other hand, Park et al. reported four imaging characteristics of IDH-wt tumors: non-lobar location, the proportion of enhancing tumor of >33%, multifocal/multicentric distribution, and poor definition of non-enhancing margins [5]. Therefore, it is assumed that gliomas showing aggressive behavior on MRI may be IDH-wt.

Immunohistochemistry and sequence tests are the gold standards for examining IDH status. However, the detection of 2-hydroxyglutarate in magnetic resonance spectroscopy (MRS) has shown a 90% or higher sensitivity and specificity for predicting IDH-mut [6]. This test is non-invasive but requires special software such as LCModel (L.A. Systems, Inc., Tokyo, Japan). In contrast, magnetic resonance spectroscopy (MRS) using standard markers such as choline (Cho), creatine (Cr), and N-acethylaspartate (NAA) is universally used and highly versatile in community hospitals. However, only one published study has sought to determine IDH status by using standard MRS for gliomas to the best of our knowledge [7]. Methionine (MET) positron emission tomography (PET) studies have shown a significantly higher accumulation of MET in IDH-wt gliomas than in IDH-mut gliomas [7-10].

This study aimed to establish the method of radiological prediction of IDH mutational status using MRI, MRS, and MET-PET and to verify their pathological implications.

This article was previously posted to the Research Square preprint server on May 26, 2022.

## Materials and methods

Study population

We retrospectively enrolled 75 patients who underwent MET-PET and MRS on the same day at our institution between January 2013 and June 2020. All patients had known pathological diagnoses of grade II or III astrocytomas, based on the 2016 WHO classification [11]. Three neurosurgeons (YI, KM, and HY) independently assessed the patterns observed via fluid-attenuated inversion recovery (FLAIR) or T2-weighted imaging to differentiate the group with a clear boundary between the tumor and the normal brain and the group with an unclear boundary. At the time of imaging assessment, the reviewers established three categories for classifying tumors: clear boundary, unclear boundary, or neither. Assessments were based solely on imaging, as physicians were blinded to clinical data such as patient name, age, sex, and histological diagnosis. MRI was more appropriate than MET-PET for assessing the boundary status of the lower-grade astrocytoma because they exhibit low accumulation of MET.

From this initial cohort of 75 patients, we sought to analyze only the outcomes of patients for whom all three reviewers or two of the three reviewers could agree on tumor classification. The physicians’ independent assessments aligned in 44 of the 75 (58.7%) cases studied. Specifically, their assessments aligned as follows: 14 patients were classified as being in the clear boundary group, and 30 patients were classified as being in the unclear boundary group. In 10 (13.3%) cases, two of the three physicians agreed on classification, and the remaining physician could not decide; of these 10 patients, the clinicians classified seven patients as being in the clear boundary group and three patients as being in the unclear boundary group. From this cohort of 54 patients, we further excluded seven patients with an unknown IDH status; thus, 47 cases (24 men, 23 women; average age = 47.0 years; age range = 19-89 years) were ultimately included in our analysis, 

Of these 47 cases, we noted 17 cases of grade II astrocytoma and 30 cases of grade III astrocytoma, 23 cases of IDH-mut, and 24 cases of IDH-wt (Table 1). There were 18 patients in the clear boundary group and 29 in the unclear boundary group (Table 1). The average age was significantly higher for the IDH-wt group than for the IDH-mut group (P=0.005) (Table 1). No significant differences existed in sex and WHO grade between the IDH status groups; however, the IDH status and MRI findings for boundary status were significantly different (P<0.001; Table 1).

**Table 1:** Summary of cases IDH: isocitrate dehydrogenase; WHO: World Health Organization; MRI: magnetic resonance image

	IDH-wildtype	IDH-mutant	p-value
No. of cases	24	23	
Age (mean±SD)	54.2±17.5	39.4±13.3	0.005
Sex (male / female)	14/10	10/13	0.39
WHO grade			0.55
grade II	10	7	
grade III	14	16	
MRI finding			<0.001
unclear boundary	21	8	
clear boundary	3	15	
Spreading tumor ratio (%)	20.7±14.0	11.1±8.4	0.008

PET

PET was conducted prior to MRI. Eminence STARGATE (Shimadzu Corporation, Kyoto, Japan) was used, which was equipped with a three-dimensional acquisition system (Shimadzu Corporation) that provided 99 transaxial images at 2.65 mm intervals. The in-plane spatial resolution (full width at half-maximum) was 4.8 mm, and the scans were conducted in three-dimensional mode. MET was injected intravenously at 3.5 MBq/kg through the cubital vein. 

During PET data acquisition, head position was corrected using laser beams projected onto ink marks drawn on the forehead, and images were reconstructed using an ordered subset expectation-maximization algorithm. Tracer accumulation in the region of interest (ROI) was analyzed using the standard uptake value (SUV), defined as the activity concentration in the ROI at a fixed time point divided by the injected dose and then normalized to the patient’s weight. The tumor-to-normal region (T/N) ratio of MET was calculated by dividing the maximum SUV of the tumor by the mean SUV of the contralateral normal frontal cortex. The ROI for the maximum tumor SUV was selected based on the areas with the highest tracer accumulation. The maximum tumor SUV was used instead of the mean SUV for the tumor to minimize the effect of tumor heterogeneity. Because of high and unexplained intersubjective SUV variability, we used the T/N ratio instead of the absolute SUV. 

Coregistration of PET and MRI was conducted using the Dr. View image analysis software package (AJS, Tokyo, Japan). In this study, PET and MRI fusion images are referred to as MET-PET.

MRI

We used the Achieva 3.0T TX QD MRI system (Philips, Amsterdam, Netherlands) for transaxial T1-weighted imaging (T1WI; repetition time (TR), 2200 ms; inversion time (TI), 950 ms; echo time (TE), 9.5 ms; field of view (FOV), 230 ×230 mm^2^; matrix, 512×512); T2WI (TR, 4000 ms; TE, 90 ms; FOV, 230×230 mm^2^; matrix, 512×512); and FLAIR imaging (TR, 8000 ms; TI, 2400 ms; TE,120 ms; FOV, 230×230 mm^2^; matrix, 512×512). The slice thickness was 5 mm with a 1-mm slice gap. A gadolinium-based contrast agent, gadoteridol (Eisai, Japan), was injected intravenously at 0.1 mL/kg body weight for contrast-enhanced studies which we performed with the same protocol as T1WI.

To quantify the extent of the lesion, we measured the area of the hyperintense lesion on the FLAIR images and the area of the entire brain in the same cross-section using ImageJ software (U.S. National Institutes of Health, Bethesda, MD, USA). We considered the area ratio as the spreading tumor ratio. For the T2/FLAIR image used for the reviewers’ assessment, we selected the same slice as the cross-section showing the highest accumulation of MET in the tumor on MET-PET.

MRS

Proton MRS was conducted before injection of gadolinium agent simultaneously with conventional MRI using the single-voxel point-resolved spectroscopy technique with a TR of 2000 ms and TE of 288 ms. The total acquisition time required to obtain these parameters, including scanner adjustments, was <5 minutes. A cubic voxel with a side length of 2.0 cm was manually placed on the lesion, for which MET-PET showed the highest accumulation, avoiding hemorrhagic lesions and cysts as much as possible. Spectra were generated using an internal scanner software, thereby providing automatic peak assignment and ratio calculations. The NAA/ Cr, Cho/Cr, and Cho/NAA peak ratios were recorded. Cr was used as the benchmark.

Pathological evaluation

We prepared formalin-fixed paraffin-embedded sections of labeled tissue for histology. The specimens underwent hematoxylin & eosin (H&E) staining and immunohistochemistry to determine IDH status using an anti-IDH1R132H monoclonal antibody (1:20; Dianova; Hamburg, Germany 1:20). Immunoreaction was considered positive when the tumor cells showed strong and diffuse staining for IDH1R132H.

The specimens were also stained with an anti-Ki-67 antibody (1:100; Dako, Tokyo, Japan) to evaluate tumor proliferation. The Ki-67 labeling index was visually quantified by counting the number of immunopositive nuclei in areas with the highest Ki-67 immunoreactivity as the percentage of Ki-67 positive cells per 1000 tumor cells.

The antigen was retrieved in an autoclave (121°C for 15 min). We used the Envision kit (Dako) as a source of secondary antibodies and 3,3-diaminobenzidine as the chromogen.

Statistical analysis

We used Fisher’s exact test to assess associations between IDH status and sex, WHO grade, and tumor-brain boundary status, and the Mann-Whitney U test to compare the spreading tumor ratio, MRS parameters, MET T/N ratio, and Ki-67 labeling index between IDH-wt and IDH-mut groups. 

Statistical significance was set at P<0.05. Receiver operating characteristic (ROC) analysis was conducted to determine the optimal thresholds of the Cho/Cr, Cho/NAA, and MET T/N ratios for discriminating IDH-wt from IDH-mut and the area under the curve. We determined the optimal sensitivity and specificity from the highest sum of the receiver operating characteristics (ROC) curve. R software (R Project for Statistical Computing, Vienna, Austria, version 4.0.3) was used for all statistical analyses.

## Results

The spreading tumor ratios were significantly higher for the unclear boundary group than for the clear boundary group (21.2±12.5 vs. 6.6±3.2, P<0.001), but the correlation between boundary status and tumor grade was not significant (P=0.99). Therefore, this finding confirmed that our reviewers’ imaging-based classifications of the clear and unclear boundary groups were objectively different. Spreading tumor ratios were significantly higher for the IDH-wt group (20.7±14.0) than for the IDH-mut group (11.1±8.4) (P=0.008) (Table 1). The association between tumor grade and IDH status was not significant (P=0.55). By contrast, the association between boundary status and the IDH status was significant (P<.001); the unclear boundary group contained significantly more IDH-wt cases, whereas the clear boundary group contained significantly more IDH-mut cases (Table 1).

The Cho/Cr, Cho/NAA, and MET T/N ratios for the IDH-wt and IDH-mut groups showed significant differences (P=0.02, P=0.047, and P=0.02, respectively); however, the NAA/Cr ratio and Ki-67 labeling index showed no significant difference between the two groups (Table 2). All MRS parameters and the Ki-67 labeling index showed no significant differences between the unclear and clear boundary groups. Only the MET T/N ratio was significantly different between the two groups (Table 2).

**Table 2:** The test values by boundary status on MRI and IDH status MRI: magnetic resonance imaging; IDH: isocitrate dehydrogenase; FLAIR: fluid-attenuated inversion recovery; NAA: N-acethylaspartate; Cr: creatine; Cho: choline; LI: labeling index; MET: methionine; T/N: tumor to the normal region

	Classification based on T2/FLAIR image		p-value	IDH status		p-value
	unclear boundary	clear boundary		IDH-wildtype	IDH-mutant	
NAA/Cr ratio	0.87±0.44	0.86±0.41	0.7	0.90±0.38	0.80±0.43	0.31
Cho/Cr ratio	1.96±0.79	2.11±0.45	0.16	1.75±0.66	2.18±0.55	0.02
Cho/NAA ratio	3.38±3.37	3.28±2.40	0.59	2.45±1.97	4.27±3.78	0.047
MET T/N ratio	2.49±0.99	1.80±0.86	0.001	2.51±1.04	1.94±0.87	0.02
Ki-67 LI	7.49±8.38	4.59±4.89	0.37	8.31±9.89	4.05±2.32	0.76

**Figure 1:**
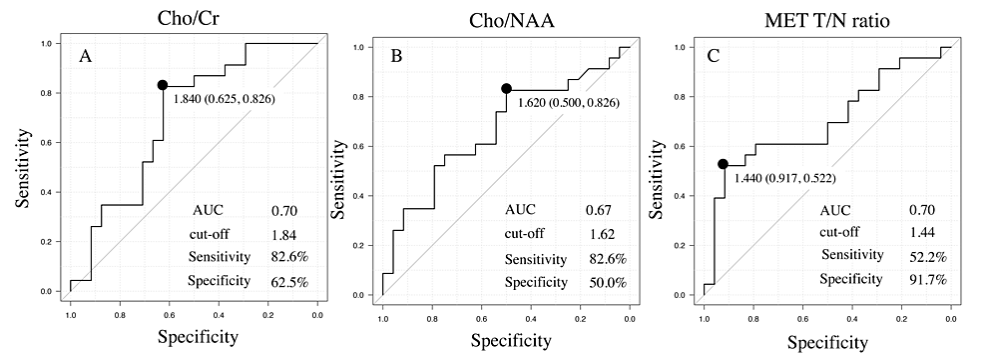
The ROC curves of Cho/Cr, Cho/NAA, and MET T/N ratio to differentiate IDH-wt from IDH-mut cases. The ROC curves for the ratios of (A) Cho/Cr, (B) Cho/NAA, and (C) MET T/N for differentiating IDH-wt from IDH-mut cases. The area under the curve, cut-off, sensitivity, and specificity values are shown for each ratio. The closed circle indicates the point where the sum of the sensitivity and specificity is at its maximum. ROC: receiver operating characteristics; MET: methionine; T/N: tumor-to-normal region; IDH: isocitrate dehydrogenase; wt: wildtype; mut: mutant; AUC: area under the curve; Cho: choline; Cr: creatine; NAA: N-acethylaspartate

We conducted ROC curve analyses to discriminate IDH-wt from IDH-mut based on the Cho/Cr, Cho/NAA, and MET T/N ratios, which showed significant differences between IDH-wt and IDH-mt statuses. The cut-off value for the Cho/Cr ratio was 1.84, with sensitivity and specificity of 82.6% and 62.5%, respectively (Figure 1A). The cut-off value for the Cho/NAA ratio was 1.62, with sensitivity and specificity of 82.6% and 50.0%, respectively (Figure 1B). The cut-off value for the MET T/N ratio was 1.44, with sensitivity and specificity of 52.2% and 91.7%, respectively (Figure 1C). The area under the curve for the Cho/Cr, Cho/NAA, and MET T/N ratios was 0.70, 0.67, and 0.70, respectively (Figure 1).

**Figure 2:**
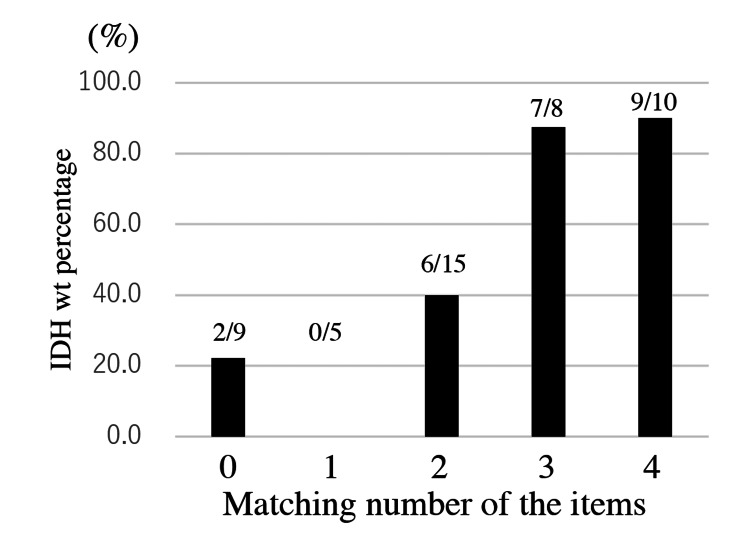
The bar graph showing the predictive rate of IDH-wt. The bar graph shows the predictive rate of IDH-wt for combinations of the following criteria: Cho/Cr ≤1.84, Cho/NAA ≤1.62, T/N ratio ≥1.44, and unclear boundary on MRI. The figure in the bar indicates the number of IDH-wt cases per number of cases diagnosed, based on each of the criteria. IDH: isocitrate dehydrogenase; wt: wildtype; mut: mutant; Cho: coline; Cr: creatine; NAA: N-acethylaspartate; MRI: magnetic resonance imaging

We then calculated the predictive accuracy for IDH-wt from the combination of the following four items: “unclear boundary,” “Cho/Cr ratio ≤1.84,” “Cho/NAA ratio ≤1.62,” and “MET T/N ratio ≥1.44”. The accuracies for predicting IDH-wt for the conditions of (1) no match, (2) one match, (3) two matches, (4) three matches, and (5) matches for all four items were 22.2%, 0%, 40.0%, 87.5%, and 90.0%, respectively (Figure 2).

Representative cases

Case 1

**Figure 3:**
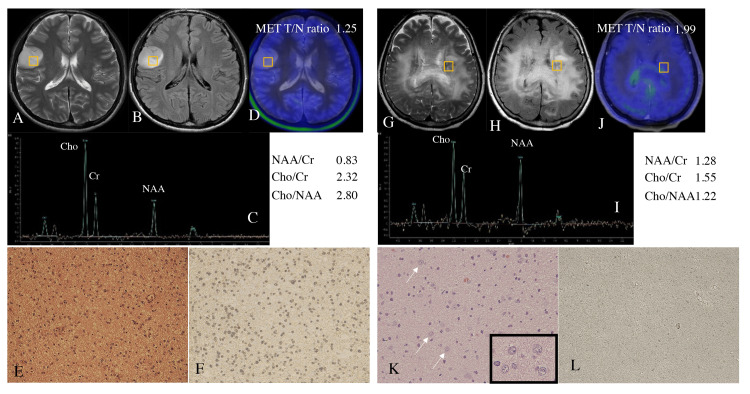
Representative cases (A–F) Representative Case 1. (G–L) Representative Case 2. (A and G) T2WI. (B and H) FLAIR images. (C and I) MRS findings. (D and J) MET-PET images. The yellow boxes in these images indicate the ROI for MRS. (E and K) Findings with H&E staining. (F and L) Immunohistochemistry for IDH1R132H shows (F) immune-positivity (i.e., IDH-mut) and (L) immune-negativity (i.e., IDH-wt). The arrows and the inset in K show ganglion-like cells. The NAA/Cr, Cho/Cr, Cho/NAA, and MET T/N ratios are shown for each representative case. The original magnifications are ×100 for E, F, K, and L and ×200 for the inset in K. T2WI: T2-weighted image; FLAIR: fluid-attenuated inversion recovery; MRS: magnetic resonance spectroscopy; ROI: region of interest; H&E: hematoxylin & eosin; MET: methionine; PET: positron emission tomography; IDH: isocitrate dehydrogenase; wt: wildtype; mut: mutant; T/N: tumor-to-normal region; NAA: N-acethylaspartate; Cho: choline; Cr: creatine

A 19-year-old man presented with convulsions. MRI revealed a brain tumor in the right frontal lobe, which showed low signal intensity on T1WI and high signal intensity with clear boundaries on T2WI (Figure 3A) and FLAIR imaging (Figure 3B). The lesion showed no contrast enhancement. The spreading tumor ratio was 5.3%. MRS showed NAA/Cr, Cho/Cr, and Cho/NAA ratios of 0.83, 2.32, and 2.80, respectively (Figure 3C). The MET T/N ratio was 1.25 (Figure 3D). H&E staining showed cell proliferation with nuclear atypia, but no strong endothelial proliferation or central necrosis was observed (Figure 3E). The Ki-67 labeling index was 7%. While immunohistochemistry for IDH1R132H showed diffuse positive staining (Figure 3F), fluorescence in situ hybridization analysis for the loss of heterozygosity of 1p and 19q revealed no co-deletion. These results led to a final histological diagnosis of anaplastic astrocytoma, IDH-mut. The patient returned to his social life without neurological deficits and showed no post-craniotomy recurrence during five years of follow-up. 

Case 2

A 70-year-old woman experienced gait disturbance due to bilateral lower-limb weakness. MRI showed a nonenhanced tumor bilaterally spreading to the periventricular white matter with unclear boundaries (Figure 3G and 3H). The spreading tumor ratio was 55.2%. The NAA/Cr, Cho/Cr, and Cho/NAA ratios were 1.28, 1.55, and 1.22, respectively (Figure 3I). The MET T/N ratio was 1.99 (Figure 3J). These findings led to gliomatosis cerebri as the preoperative clinical diagnosis. H&E staining showed scattered large ganglion cells and atypical glial cells, but the cell density was not high (Figure 3K). While immunohistochemistry showed IDH1R132H-negativity (Figure 3L), the Ki-67 labeling index was 6.5%. The final histological diagnosis was diffuse IDH-wt grade II/III astrocytoma with a gliomatosis cerebri pattern. In the present study, we treated this case as grade III. The patient received 40 Gy whole-brain irradiation with temozolomide. She required full assistance seven months after the biopsy due to quadriplegia and confusion.

## Discussion

Several reports exist regarding the prediction of IDH-mut based on detecting 2-hydroxyglutarate [6,12,13]. In addition, in one report [7], the IDH status was predicted using the combination of MRS and MET-PET. We did not have special software to detect 2-hydroxyglutarate based on MRS; therefore, we relied upon the standard and versatile MRS parameters Cho, Cr, and NAA. We have shown that the Cho/Cr, Cho/NAA, and MET T/N ratios are significantly different between the IDH-mut group and the IDH-wt group, while the ROC curve does not necessarily have a high AUC for each. Therefore, it is necessary to predict the IDH status using the boundary status in MRI, MRS, and MET-PET in combination. Our study aimed to establish a method for predicting the IDH status and determine the pathological implications of the MRS and MET-PET findings for predicting IDH status by comparing the unclear and clear boundary groups using preoperative MRI findings. 

This study revealed more IDH-wt lower-grade astrocytic gliomas in the unclear boundary group than in the clear boundary group. This finding was supported by the significantly higher spreading tumor ratio in the IDH-wt group than in the IDH-mut group. By contrast, Baldock et al. analyzed the MRIs of 172 patients with contrast-enhanced gliomas and found no significant difference in the dispersal index between the IDH-wt and IDH-mut groups [14]. However, we observed significantly more cases of IDH-wt in the unclear boundary group, with a significantly higher Ki-67 labeling index than in the clear boundary group. Accordingly, clinicians should consider a tumor with an unclear boundary as invasive and aggressive.

Gliomatosis cerebri was excluded from the 2016 WHO classification because it comprises a genetically and epigenetically heterogeneous group of diffuse gliomas [15]. We encountered seven cases that met the criteria of the 2007 WHO classification [16], which defined them as extensively infiltrative diffuse gliomas involving at least three cerebral lobes. Kwon et al. performed a molecular classification of 89 cases of gliomatosis cerebri and showed that 73% were IDH-wt [17]. Of the 29 cases (IDH-mt 9 cases and IDH-wt 20 cases) that could be followed, the malignant transformation was observed in four cases (44%) in the former and 17 cases (85%) in the latter cases. Thus, the infiltrative patterns on MRI are a significant radiological marker for IDH-wt, implying a poor prognosis.

Histological findings of necrosis and microvascular proliferation are reportedly more frequent in gliomatosis cerebri with IDH-wt than those with IDH-mut [18]. However, H&E findings cannot generally predict IDH status. Determining the mechanism of invasion depends on IDH status: For IDH-mut cases, the expression of oncogenes such as the platelet-derived growth factor receptor was associated with glioma formation [19] and the migratory capacity of glioma cells [20,21]. For IDH-wt cases, the receptor tyrosine kinase-phosphatidylinositol-3/serine-threonine protein kinase-mechanistic target of the rapamycin pathway has been reported to promote invasion [22]. Notably, the six-transmembrane epithelial antigen of prostate 3 is significantly more expressed in IDH-wt tumors than in IDH-mut tumors and is involved in the invasiveness of gliomas [23]. Regardless of the IDH status, the tumors all have molecular mechanisms involved in infiltration.

In the present study of lower-grade astrocytoma with marked invasiveness, the Cho/Cr and Cho/NAA ratios were significantly lower for IDH-wt cases. Cho is a metabolic marker of cell membrane density and integrity [24] and may be elevated because of increased membrane synthesis in rapidly dividing tumor cells [25]. A surprising finding was that the Cho/Cr ratio was significantly lower in the IDH-wt group, although the MET T/N ratio and Ki-67 labeling index were significantly higher in the IDH-wt group than in the IDH-mut group. This finding may be because of low cell density, as observed in the pathological findings of Case 2. These results for the Cho/Cr and MET T/N ratios were consistent with reports by Kebir et al. [7] and Zhou et al. [8].

NAA is an internal neuronal marker that parallels the relatively constant neuronal cell density [26]. One report [27] showed a tendency toward a higher NAA peak for grade II gliomas than high-grade glioma. The NAA density decreases with increasing glioma grading, and the Cho/NAA ratio is a sensitive method for detecting differences in tumor growth [28]. In our data, the NAA/Cr ratio showed no significant difference between the IDH-wt and IDH-mut groups. However, the Cho/NAA ratio was significantly different between the two groups because the Cho/Cr ratio was significantly lower for the former than for the latter, and the NAA/Cr ratio was slightly higher for the former. Thus, we showed that the Cho/Cr and Cho/NAA ratios were significantly lower for the IDH-wt group than for the IDH-mut group. Clinicians should consider marked invasive glioma on preoperative MRI with these MRS findings as a case of IDH-wt.

The cut-off values for the Cho/Cr, Cho/NAA, and MET T/N ratios for differentiating IDH-wt from IDH-mut cases in the present series obtained by ROC analyses were 1.84, 1.62, and 1.44, respectively. However, these values were obtained from a limited series; therefore, care must be taken to ensure versatility and reproducibility. A higher diagnostic rate of IDH-wt was associated with meeting more of the following criteria: Cho/Cr ≤1.84, Cho/NAA ≤1.62, T/N ratio ≥1.44, and an unclear boundary (Figure 2). Therefore, these items seem reasonable for predicting IDH status. Representative Case 2 was expected to be IDH-wt because all the criteria mentioned above were met. We believed that the findings showing multiple ganglion-like cells with sparse cell density affected the decrease in Cho/NAA and Cho/Cr ratios in this case.

The WHO Classification of Tumors, 5th edition [29], was released in 2022. It classifies all IDH-wt adult gliomas as grade 4. However, none of the patients in this study could be classified according to this version because they did not undergo grade 4-confirming molecular testing such as telomerase reverse transcriptase promoter mutation, epidermal growth factor receptor amplification, and chromosome 7 gain/chromosome 10 loss. Also, a data collection period of seven years can significantly bias retrospective analysis. These were limitations of this retrospective study.

## Conclusions

A decrease in the Cho/Cr or Cho/NAA ratio in an intra-axial brain tumor with unclear boundaries suggests IDH-wt. Due to its high infiltration, the Cho/Cr ratio is likely underestimated because the tumor cell density is sparse in an IDH-wt tumor. However, we considered that the neuronal cells in the IDH-wt group had a lower Cho/NAA ratio than those in the IDH-mut group. Although our study does not correspond to the new WHO classification, 5th edition, the pathological infiltrative nature of IDH-wt could be supported by MRS and MET-PET information. This diagnostic method of combining MRI, MRS, and MET-PET helped predict the IDH status of lower-grade astrocytoma. We need to verify this method's prediction of IDH status through a future prospective study using the new WHO classification, 5th edition.
